# The association between osteoporosis and frailty: a cross-sectional observational study and mendelian randomization analysis

**DOI:** 10.1186/s13018-024-04875-w

**Published:** 2024-07-09

**Authors:** Zhiqiang Que, Yilong Lin, Dingqiang Chen, Keyi Xiao, Wenbin Xu, Naikun Sun, Qingmo Yang, Gang Rui

**Affiliations:** 1 Department of Orthopedics, The First Affiliated Hospital of Xiamen University, School of Medicine, Xiamen University, Xiamen, China; 2 Department of Breast Surgery, The First Affiliated Hospital of Xiamen University, School of Medicine, Xiamen University, Xiamen, China; 3 The School of Clinical Medicine, Fujian Medical University, Fuzhou, China

**Keywords:** Osteoporosis, Frailty, National Health and Nutrition Examination Survey, Mendelian randomization

## Abstract

**Background:**

Osteoporosis and frailty are two common features in the elderly population. Despite many review articles mentioning the association between osteoporosis and frailty, there is a lack of original research directly investigating their relationship. Therefore, this study was conducted to examine the correlation between osteoporosis and frailty.

**Methods:**

We conducted a cross-sectional study using data from the National Health and Nutrition Examination Survey (NHANES), using logistic regression analysis to assess the association of osteoporosis with the frailty index. In addition, we further explored the causal relationship between them using Mendelian randomization (MR) study.

**Results:**

In the cross-sectional study, 19,091 non-frailty participants and 5878 frailty participants were included in this study. We observed a significant positive association between osteoporosis and frailty after adjusting for demographic characteristics, body mass index (BMI), smoking, and alcohol use (OR = 1.454, 95% CI [1.142,1.851], *P* = 0.003). Moreover, the MR study showed a bidirectional causal relationship between osteoporosis and frailty. When osteoporosis was used as an exposure factor, the frailty pooled OR value calculated utilizing the inverse variance weighted (IVW) method was 2.81 (95% CI [1.69, 4.68], *P* = 6.82 × 10^− 5^). When frailty was used as an exposure factor, the OR value calculated using the IVW method was 1.01 (95% CI [1.00,1.01], *P* = 3.65 × 10^− 7^).

**Conclusions:**

Osteoporosis was positively correlated with frailty, and the results remained robust after adjusting for covariates. Further, MR studies have shown a bidirectional causal relationship between osteoporosis and frailty.

**Supplementary Information:**

The online version contains supplementary material available at 10.1186/s13018-024-04875-w.

## Introduction

Osteoporosis is a progressive systemic bone disease characterized by low bone mass, deterioration of bone tissue micro-structure, and increased bone fragility [1]. It is diagnosed using the T-score calculated from bone mineral density (BMD), where osteoporosis is confirmed when the BMD is less than or equal to 2.5 standard deviations (SD) (T-score ≤ -2.5) compared to normal young adults [2]. A variety of factors are closely related to osteoporosis, including age, sex, ethnicity, BMI, smoking, alcohol consumption, diabetes, hyperthyroidism, premature menopause (< 45 years), and glucocorticoid use [3, 4]. Approximately 200 million women globally are afflicted by osteoporosis, with osteoporotic fractures affecting 30% of women and 20% of men aged 50 and above [5–7]. As the global population ages, the number of incident osteoporosis-related fractures is expected to increase by 310% in 2050 or earlier [8]. The resulting economic burden is also significant, spending about $17.9 billion in the US and £4 billion in the UK per year, respectively [9–11]. Therefore, the early identification and treatment of osteoporosis is important.

Frailty is a state in which individuals face a higher risk of negative outcomes due to the decline in physiological reserves and functioning of multiple organ systems associated with aging [12]. Research indicates that 25%–50% of individuals aged 85 and above experience frailty [13, 14]. It cannot be overlooked due to its association with increased healthcare utilization, disability, and mortality among individuals in frail states [15]. The high prevalence of frailty and its substantial social burden underscore the significance of addressing this issue. While numerous review articles have highlighted the connection between frailty and osteoporosis, the direct investigation of their relationship is limited in original studies, leading to conflicting findings across various research studies [16, 17]. Therefore, the current understanding of the relationship between osteoporosis and frailty is insufficient and this topic is still worth exploring.

National Health and Nutrition Examination Survey (NHANES) is a program to evaluate the health and nutritional status of both adults and children in the US [18]. It combines interviews and physical examinations to collect data, providing a vast array of reliable information for determining the prevalence of major diseases and risk factors for diseases. Mendelian randomization (MR) analysis is an epidemiological method that utilizes genetic variations as instrumental variables (IVs) to evaluate the causal association between exposure factors and outcome events. MR analysis offers a distinct advantage in establishing an unbiased causal relationship between exposure and outcomes.

Hence, we undertook a cross-sectional study to explore the association between osteoporosis and frailty using the NHANES data (2005–2010, 2013–2014, 2017–2018). The MR analysis was used to investigate the bidirectional causal effect of osteoporosis on frailty. The goal of this study is to provide reliable epidemiological evidence on the relationship between osteoporosis and frailty, raise awareness of the early recognition of these two conditions in clinical practice, and even accelerate the discovery of effective strategies to prevent osteoporosis and frailty.

## Methods

### The cross-sectional analysis

#### Data source

Data for this cross-sectional study are from NHANES (2005–2010, 2013–2014, 2017–2018). The survey collects demographic information, dietary habits, examination results, laboratory data, and questionnaire responses annually from a nationally representative sample of around 5000 individuals and publishes the results biennially. The study protocol received approval from the Ethics Review Board of the National Center for Health Statistics. All participants provided written informed consent. This study followed the Reporting on Strengthening Observational Studies in Epidemiology (STROBE) reporting guidelines.

#### Ascertainment of osteoporosis

Dual-energy x-ray absorptiometry (DXA) scans of the proximal femur have been performed at the NHANES mobile examination center (MEC) since 2005. However, femoral BMD assessments were not performed during the 2011–2012 and 2015–2016 cycles. Therefore, data from five NHANES cycles were utilized in this study: 2005–2010, 2013–2014, and 2017–2018. According to the diagnostic criteria of the WHO [2], osteoporosis is defined as a BMD value equal to or less than 2.5 SD compared to a normal young population. This criterion was applied to the femoral neck and lumbar region to diagnose osteoporosis.

#### Ascertainment of frailty

According to previous research [19], frailty index (FI) is computed by dividing the accumulated deficits of a participant by the overall number of items assessed. When the FI > 0.21, the individual is defined as frailty. The evaluation indicators involved 7 aspects: **cognition** (experience confusion/memory problems), **dependence** ( managing money difficulty; stooping, crouching, kneeling difficulty; lifting or carrying difficulty; house chore difficulty; preparing meals difficulty; standing up from armless chair difficulty; getting in and out of bed difficulty; using fork, knife, drinking from cup difficulty; dressing yourself difficulty; standing for long periods difficulty; grasp/holding small objects difficulty; attending social event difficulty; push or pull large objects difficulty; walking for a quarter mile difficulty; walking up 10 steps difficulty), **depressive symptoms** (have little interest in doing things; feeling down, depressed, or hopeless; trouble sleeping or sleeping too much; feeling tired or having little energy; poor appetite or overeating; feeling bad about yourself; trouble concentrating on things), **comorbidities** (arthritis; thyroid problems; chronic bronchitis; cancer; congestive heart failure; coronary heart disease; angina; heart attack; stroke; blood pressure; diabetes; weak/failing kidneys; urinary leakage), **hospital utilization and access to care** (self-rated health; health now compared with 1 year ago; overnight hospital patient in past year; frequency of health care use during past year; number of prescribed medications), **physical performance and anthropometry** (body mass index; handgrip strength), and **laboratory values**(glycohemoglobin (%); red blood cell count (million cells/mL); hemoglobin (g/dL); red cell distribution width (%); lymphocyte percent (%); segmented neutrophils percent (%)), with a total of 49 indicators. Detailed grading criteria have been shown in Supplementary Table 1.

#### Covariates

Drawing upon previous literature, and clinical expertise, and considering the NHANES database accessibility, we incorporated the subsequent indicators as covariates, including age, ethnicity, educational level, marital status, poverty income ratio (PIR), BMI, smoking, and alcohol consumption. Ethnicity was categorized into non-Hispanic White, non-Hispanic Black, Mexican American, and other. The educational level was segmented into under high school, high school or equivalent, and above high school. Depending on whether the individual is living with a partner, dividing the marital status into married or living with partner and other. The other group includes the population who never married, divorced, separated, and widowed. PIR was classified as 0-1.3 PIR, > 1.3–3.5 PIR, > 3.5 PIR. BMI was divided into four levels, including underweight (< 18.5 kg/m^2^), normal (18.5–24.9 kg/m^2^), overweight (25–29.9 kg/m^2^), obese (≥ 30 kg/m^2^). Smoking was divided into three groups, including never smoking, former smoking and now smoking; Alcohol consumption was divided into five groups based on whether and to what extent alcohol is consumed, including never, former, mild, moderate, and heavy; Individuals with diabetes and taking diabetes drugs would be regarded as diabetic patients. Participants who actively respond to the questionnaire with hypertension and who are taking medication for hypertension would be considered hypertensive patients.

#### Statistical analysis

In this study, continuous variables were expressed as mean and SD, and categorical variables were presented as numbers (n) and percentages (%). The population was categorized into the non-frailty group (*N* = 19,091) and frailty group (*N* = 5878) based on FI, and the T-test and Chi-square test were employed to assess significant differences between these groups. Logistic regression models were used to evaluate the correlation between osteoporosis and frailty. In this analysis, we built four models. The crude model did not include adjustments for any covariates. Model 1 was adjusted for age, sex, ethnicity, marital status, PIR, and education level. Model 2 was adjusted for age, sex, ethnicity, marital status, PIR, education level, and BMI. Model 3 was adjusted for age, sex, ethnicity, marital status, PIR, education level, BMI, smoking and alcohol use. Further stratification analyses were performed for age, ethnicity, PIR, marital status, education level, smoking status, drinking, and BMI. All statistical analyses were conducted using R software (version 4.3.2), and significance was determined at a threshold of *p* < 0.05.

### Mendelian randomization analysis

#### Genome-wide association studies sources

The summary data of osteoporosis for this analysis were obtained from Genome-Wide Association Studies (GWAS) (ID: ebi-a-GCST90038656) [20], comprising 7751 cases and 476,847 controls, all participants were European population. The data of FI (ID: ebi-a-GCST90020053) were sourced from the UK Biobank (*n* = 164,610) and Swedish TwinGene participants (*n* = 10,616)’s GWAS meta-analysis. 175,226 participants of European descent were obtained and a total of 49 items were assessed, with a mean defect ratio of 0.129 (0.075) [21]. A total of 10,616 individuals were obtained from TwinGene, all of European ancestry. A total of 44 items were evaluated, with an average defect ratio of 0.121 (0.080).

#### Selection of genetic instrumental variables

Three assumptions [22] are met in this MR study: (1) The link between gene expression and IVs is considerable; (2) IVs are not associated with confounders; and (3) IVs only affect outcomes through exposure. For this MR analysis, first, single nucleotide polymorphisms (SNPs) strongly correlated with exposure were selected as IVs (*P* < 5.0 × 10^− 5^). Second, we excluded SNPs with linkage disequilibrium (LD) (r^2^ > 0.001, kb = 10,000). Next, the IVs related to outcome and F-statistics < 10 were excluded in this study. Through Steiger’s test, SNPs that exhibited a lack of directional consistency were identified and removed from the analysis. At last, the MR-presso method was used to find and filter the outlier IVs.

#### Statistical analysis

This MR analysis was conducted in R software (version 4.3.2) using “TwoSampleMR package” [23], “Mendelian Randomization package” [24], and “MR-PRESSO package” [25]. The inverse variance weighted (IVW) method was used as the primary analytical approach. While the IVW method offers precise estimates, it is vulnerable to pleiotropic IVs. [26]. The biggest difference between the MR Egger method and the IVW method is that the intercept term is considered in the regression, which enables the assessment of the presence of horizontal pleiotropy [27]. The weighted median (WM) method is reliable when the majority of weights (> 50%) in the analysis are derived from valid instrumental variables [28]. MR-presso method can exclude possible abnormal values, provide new adjusted results, and correct horizontal pleiotropy. Therefore, we complement the MR Egger, Weighted median, and MR Egger methods. Sensitivity analyses were performed utilizing Cochran’s Q test [29], MR-Egger intercept test, Steiger’s test, leave-one-out test, and funnel plot.

## Result

### Baseline characteristics of study participants

**Fig. 1:**
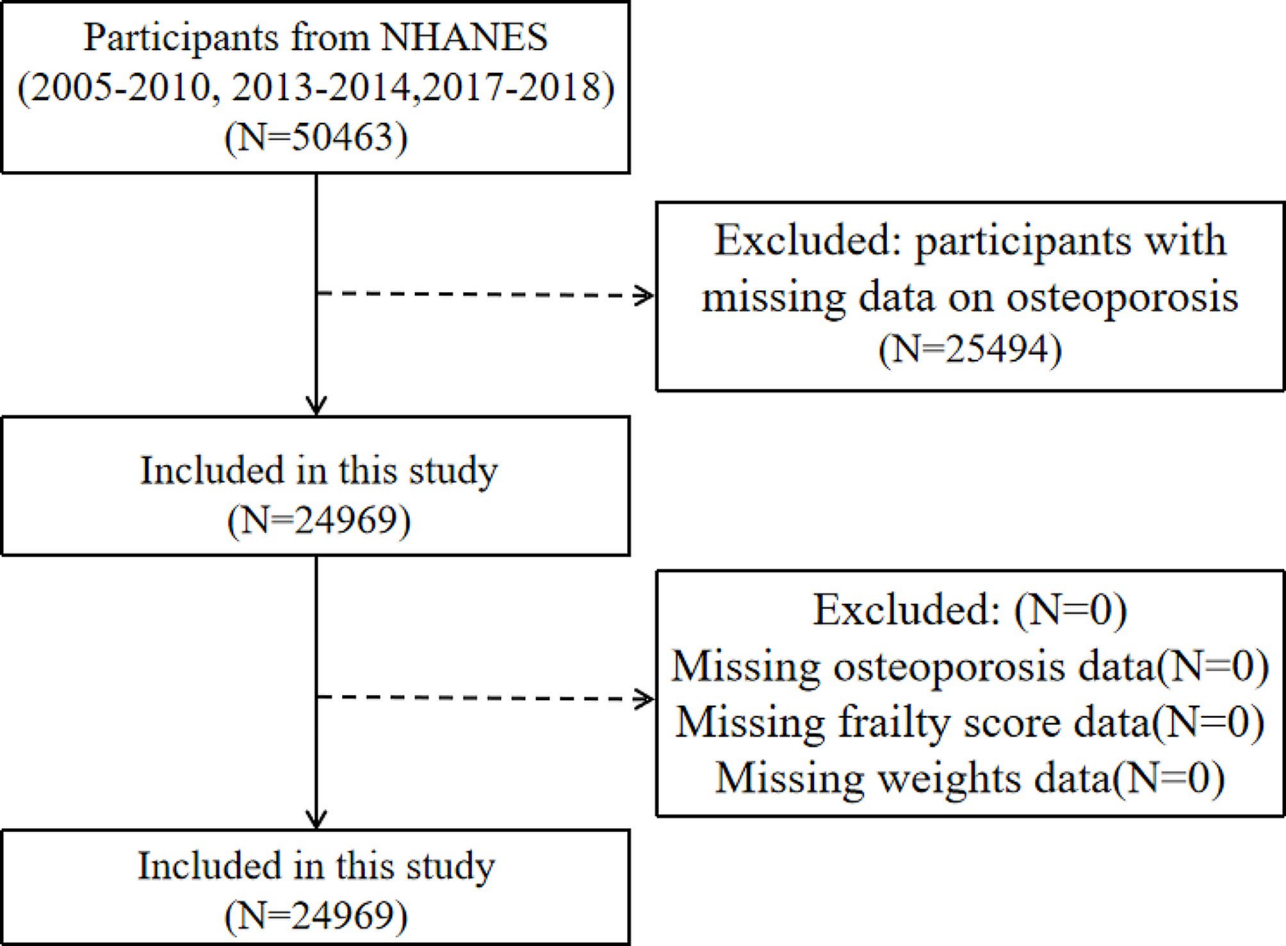
Flow chart of selecting eligible participants

In the five cycles of 2005–2010, 2013–2014, and 2017–2018, a total of 50,463 participants were included, 25,494 individuals with missing BMD data were excluded, 0 participants with missing frailty index scores were excluded, and 0 individuals with missing weight information or weighted 0 were excluded, and finally 24,969 participants, including 19,091 non-frailty participants and 5878 frailty participants, were included in the study (Fig. 1). Table 1 presents the weighted characteristics of the populations included in this study. The prevalence of frailty is relatively high in females and non-Hispanic blacks. Married or living with a partner has a lower prevalence of frailty than others. Individuals with frailty have lower income, higher age and BMI than those without frailty, and have shown a correlation with education level, alcohol consumption and smoking.

**Table 1:** Demographic and clinical characteristics of the study participants in the NHANES

Variable	Total (*N* = 24,969)	non-Frailty (*N* = 19,091)	Frailty (*N* = 5878)	*P* value
**Age**	44.91(0.31)	44.05(0.32)	48.74(0.47)	< 0.0001
**PIR**	3.06(0.04)	3.20(0.04)	2.45(0.05)	< 0.0001
**Sex**				< 0.0001
Female	12,118(49.85)	9137(48.70)	2981(54.97)	
Male	12,851(50.15)	9954(51.30)	2897(45.03)	
**Ethnicity**				< 0.0001
Non-Hispanic White	10,598(69.11)	8337(70.40)	2261(63.38)	
Non-Hispanic Black	5372(10.77)	3690( 9.47)	1682(16.57)	
Mexican American	4974( 8.51)	3957(8.68)	1017(7.75)	
Other	4025(11.61)	3107(11.46)	918(12.29)	
**Marital** status				< 0.0001
Married or living with partner	11,571(56.20)	9491(66.07)	2080(55.09)	
Other	8492(31.30)	6331(33.93)	2161(44.91)	
**Education level**				< 0.0001
Under high school	5628(13.04)	3704(10.98)	1924(22.30)	
High school or equivalent	9754(36.18)	7340(34.86)	2414(42.19)	
Above high school	9562(50.70)	8029(54.16)	1533(35.51)	
**Alcohol use**				< 0.0001
Never	2285( 8.28)	1741(10.10)	544(12.46)	
Former	3026(11.45)	2082(12.60)	944(24.34)	
Mild	5918(29.86)	4848(38.52)	1070(33.89)	
Moderate	2552(13.43)	2145(17.56)	407(14.01)	
Heavy	3333(16.02)	2823(21.22)	510(15.30)	
**Smoke**				< 0.0001
Never	9824(45.16)	8148(55.46)	1676(41.35)	
Former	4909(22.17)	3657(24.97)	1252(31.49)	
Now	3945(17.73)	2920(19.57)	1025(27.16)	
**Hypertension**				< 0.0001
No	16,329(65.50)	13,398(70.43)	2931(43.99)	
Yes	8567(34.36)	5640(29.57)	2927(56.01)	
**Diabetes**				< 0.0001
No	22,320(91.25)	17,841(94.61)	4479(76.30)	
Yes	2649( 8.75)	1250( 5.39)	1399(23.70)	
**Osteoporosis**				< 0.0001
No	23,117(93.96)	17,923(95.15)	5194(88.66)	
Yes	1852( 6.04)	1168( 4.85)	684(11.34)	
**BMI**	27.25(0.08)	27.09(0.08)	27.99(0.14)	< 0.0001

Continuous variables were presented by mean and standard deviation (SD), and categorical variables were presented with numbers(n) and percentages (%)

PIR, ratio of family income to poverty; BMI, body mass index

### Association between osteoporosis and frailty

The results of this cross-sectional study using NHANES data are shown in Table 2. The incidence of osteoporosis showed a significantly positive correlation with frailty. The OR values of crude model, model 1, model 2 and model 3 were 2.509(95% CI [2.183,2.883], *P* < 0.0001), 1.359(95% CI [1.094,1.690], *P* = 0.006), 1.654(95% CI [1.328, 2.061], *P*  < 0.0001) and 1.454(95% CI [1.142, 1.851], *P* = 0.003), respectively. In addition, stratified analyses were conducted, revealing a consistent association between osteoporosis and frailty across various strata, including ethnicity, marital status, alcohol use and smoking status. (Table 3).

**Table 2:** Multivariate logistic regression analysis of osteoporosis with frailty

Character	Crude model		Model 1		Model 2		Model 3	
	95%CI	*P*	95%CI	*P*	95%CI	*P*	95%CI	*P*
non-Frailty	ref		ref		ref		ref	
Frailty	2.509(2.183,2.883)	< 0.0001	1.359(1.094,1.690)	0.006	1.654(1.328,2.061)	< 0.0001	1.454(1.142,1.851)	0.003

Crudel model: no adjustment was made for any covariates

Model 1:adjusting for age, sex, ethnicity, marital status, PIR, education level

Model 2:adjusting for age, sex, ethnicity, marital status, PIR, education level, BMI

Model 3:adjusting for age, sex, ethnicity, marital status, PIR, education level, BMI, smoking, alcohol use

**Table 3:** Stratified associations between osteoporosis and frailty according to baseline characteristics

Character	95% CI	*P*	*P* for interaction
**Sex**			< 0.0001
Female	2.008(1.682,2.397)	< 0.0001	
Male	3.326(2.757,4.013)	< 0.0001	
**Ethnicity**			0.28
Non-Hispanic White	2.780(2.311,3.342)	< 0.0001	
Non-Hispanic Black	2.586(1.999,3.346)	< 0.0001	
Mexican American	2.153(1.796,2.581)	< 0.0001	
Other	2.267(1.590,3.233)	< 0.0001	
**Marital status**			0.117
Married or living with partner	2.160(1.717,2.717)	< 0.0001	
Other	2.801(2.138,3.669)	< 0.0001	
**PIR**			< 0.001
0-1.3 RIP	1.537(1.262,1.872)	< 0.0001	
>1.3–3.5 RIP	2.450(2.000,3.001)	< 0.0001	
>3.5 RIP	3.431(2.467,4.772)	< 0.0001	
**Education level**			0.002
Under high school	1.460(1.195,1.784)	< 0.001	
High school or equivalent	2.052(1.654,2.547)	< 0.0001	
Above high school	2.983(2.091,4.255)	< 0.0001	
**Alcohol use**			0.22
Never	2.126(1.464,3.086)	< 0.001	
Former	1.860(1.205,2.873)	0.006	
Mild	2.004(1.423,2.821)	< 0.001	
Moderate	2.658(1.329,5.318)	0.006	
Heavy	4.234(2.379,7.533)	< 0.0001	
**Smoke**			0.34
Never	2.978(2.298,3.858)	< 0.0001	
Former	2.302(1.724,3.072)	< 0.0001	
Now	2.387(1.652,3.450)	< 0.0001	
**BMI**			0.018
Underweight	1.570(1.158,2.128)	0.004	
Normal	3.014(2.369,3.835)	< 0.0001	
Overweight	2.586(2.021,3.307)	< 0.0001	
Obese	2.148(1.372,3.362)	0.001	

The non-frailty group was used as a reference

PIR, ratio of family income to poverty; BMI, body mass index

### The causal association between osteoporosis and frailty

**Fig. 2:**
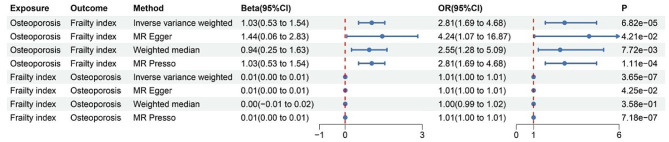
Associations between osteoporosis and frailty in two sample MR analyses

**Fig. 3:**
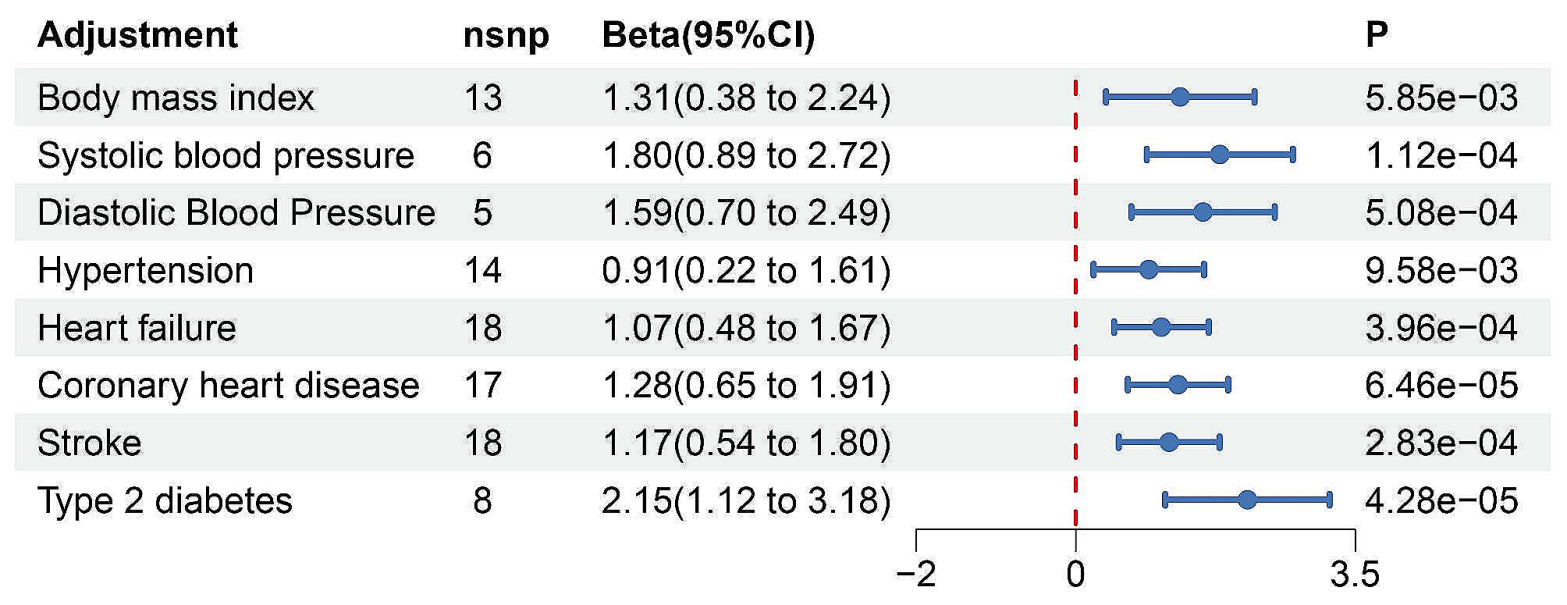
MR study of the relationship between osteoporosis and frailty after adjusting for confounders

A comprehensive bidirectional two-sample MR analysis was used to investigate the causal relationship between osteoporosis and frailty (Fig. 2). We identified a significant causal association between osteoporosis and frailty. The pooled odds ratio (OR) obtained through the IVW method was 2.81 (95% CI [1.69,4.68], *P* = 6.82 × 10^− 5^) for per SD increase in the prevalence of osteoporosis. Similar results were obtained using the MR Egger (OR = 4.24, 95% CI [1.07,16.87], *P* = 4.21 × 10^− 2^), Weighted median (OR = 2.55, 95% CI [1.28,5.09], *P* = 7.72 × 10^− 3^), and MR-presso (OR = 2.81, 95% CI [1.69,4.68], *P* = 1.11 × 10^− 4^). To explore whether the causal impact of osteoporosis on frailty is influenced by other confounding factors, we performed a multivariate MR analysis. After adjusting for these factors, including BMI, blood pressure, heart failure, coronary heart disease, stroke, and type 2 diabetes, the results remained statistically significant. (Fig. 3). In reverse Mendelian randomization, although the results from the MR analysis remain significant, the absolute value of the beta coefficient is relatively small. The osteoporosis pooled OR value calculated using the IVW method was 1.01 (95% CI [1.00,1.01], *P* = 3.65 × 10^− 7^) for per SD increasing the prevalence of frailty. Both MR Egger (OR = 1.01, 95% CI [1.00,1.01], *P* = 4.25 × 10^− 2)^ ) and MR-presso (OR = 1.01, 95% CI [1.00,1.01], *P* = 7.18 × 10^− 7^) also demonstrated significant causal associations (Fig. 2).

### Sensitivity analysis

To assess horizontal pleiotropy, we utilized Cochran’s Q test, MR-Egger intercept test, leave-one-out analyses, and funnel plot. The p-values from MR-Egger intercept tests, chosen by IVW, suggested the absence of horizontal pleiotropy as they exceeded 0.05. For the causal effect of osteoporosis on frailty, the Cochrane Q test indicated the presence of heterogeneity (Cochran’s Q-derived *P*-value = 0.04), therefore, we employed the IVW random-effects model. In contrast, there was no heterogeneity present for reverse causality. Supplementary Figure S1–S8 display scatter plots, forest plots, leave-one-out plots, and funnel plots, indicating that the estimates remained unaffected by individual SNPs and there were no violations of assumptions.

## Discussion

In this study, we conducted a cross-sectional study utilizing NHANES data to explore the association between osteoporosis and frailty. The findings of this cross-sectional study revealed a positive correlation between the incidence of osteoporosis and frailty, and that the results remained robust after adjusting for covariates and performing stratified analyses. In addition, we further explored the causal association between osteoporosis and frailty through MR study and found that there is a bidirectional causal relationship between them. To the best of our knowledge, this study represents the first investigation into the association between osteoporosis and frailty utilizing the NHANES database and MR analysis.

Although many review articles have spoken about the association between osteoporosis and frailty, few original studies directly investigate the relationship between them, and even the results of these studies are inconsistent [30–34]. A previous cohort study involving 405 participants concluded that the association between osteoporosis alone and frailty was weak and that the likelihood of frailty was only higher when sarcopenia and osteoporosis coexisted [35]. The same findings were evident in another cross-sectional study that included 250 participants [32], indicating that the association between severe osteopenia/osteoporosis and frailty in older women in the community did not reach statistical significance. However, when sarcopenia and severe osteopenia/osteoporosis coexisted, the association significantly strengthened. The participants in both studies were from the community, which may introduce cohort bias. Additionally, the relatively small sample size could impact the interpretation of the findings. Nevertheless, some studies provide evidence for a notable association between osteoporosis and frailty. Liu et al. [36] discovered an association between frailty and lower BMD, even after controlling for age, sex, and functional status. In addition, another cross-sectional study of postmenopausal women similarly showed that older adults with osteoporosis were at greater risk of frailty syndrome, with more than 75% accuracy in predicting frailty using osteoporosis [30]. However, there were some differences in the definition of frailty between these studies and ours, using Fried’s standard definition, which included exhaustion, weakness, slowness, physical inactivity, and weight loss. In this study, we defined frailty using FI, assessing 49 items involving seven aspects, including cognition, dependence, depressive symptoms, comorbidities, hospital utilization and access to care, physical performance and anthropometry, and laboratory values, which were defined as frailty when FI > 0.21. The criteria for defining frailty have an impact on the results of association [37]. Furthermore, all these studies were limited to cross-sectional designs and did not explore the causal relationship between osteoporosis and frailty further.

Our results from the cross-sectional study are in line with the MR study, indicating a significant correlation between osteoporosis and frailty. Both osteoporosis and frailty are age-related diseases, accompanied by other common features relating to age, such as muscle loss, weight loss, physical activity, falls, cognitive decline, etc., which may also strengthen the relationship between the two to some extent [29]. Some fundamental research have suggested the presence of shared pathophysiological mechanisms between osteoporosis and frailty, such as endocrine disruption and increased pro-inflammatory factors [38]. Hormones such as testosterone, estrogen, growth hormone, insulin-like growth factor 1 (IGF-1), vitamin D, and cytokines fluctuate similarly in populations with osteoporosis and frailty [38]. Estrogen and IGF-1 decrease with age, mediating the activation of osteoclast cytokines, subsequently promoting bone resorption, ultimately leading to osteoporosis [39], and by mediating elevated catabolic cytokine levels, promoting decreased muscle mass and strength, ultimately leading to frailty [40]. This elucidates why the association between osteoporosis and frailty is more significant under conditions of advanced age and sarcopenia. Chronic inflammation is one of the key factors that lead to frailty [12], similarly, the process of bone loss is inseparable from inflammation [41]. Estrogen and androgens are important inhibitors of interleukin-6 (IL-6) gene expression [42]. As the withdrawal of these sex hormones occurs, the amount of IL-6 gradually increases, and it promotes bone loss by enhancing osteoclast activity. A study has shown that the inflammatory marker C-reactive protein is also associated with frailty and osteoporosis [40]. In addition, osteoprotein, a powerful marker of osteoclast activity, was significantly elevated in patients with both osteoporosis and frailty [43, 44]. The common pathophysiological process provides a reasonable explanation for the two-way causal relationship between osteoporosis and frailty.

Some of the advantages of our research are as follows. First, the data for the cross-sectional study were obtained from the NHANES database, and we integrated samples from five cycles to obtain a large sample that was broadly representative. Second, we adjusted for covariates including demographic characteristics, BMI, smoking, and alcohol use, and performed stratified analysis to make the results more reliable. Finally, we further explored the causal relationship between osteoporosis and frailty by employing MR analyses. Still, there are some shortcomings to this study. First, most of the NHANES data we used are from questionnaires based on participants’ memory, and there will inevitably be some recall bias. Second, the absence of demographic data on osteoporosis in the MR study precluded us from conducting a subgroup analysis. Finally, additional basic experimental validations are necessary to affirm the association between osteoporosis and frailty, consequently enabling the prediction of future frailty risk based on the presence of osteoporosis.

## Conclusions

This study revealed a positive correlation between osteoporosis and frailty, with the findings remaining robust even after adjusting for various covariates. An additional MR analysis revealed a bidirectional causal relationship between osteoporosis and frailty. The current study offers a solid foundation for the concurrent management of osteoporosis and frailty. Nonetheless, further research is essential to delve deeper into the shared pathophysiological mechanisms between these two conditions.

### Electronic supplementary material

Below is the link to the electronic supplementary material.

Supplementary Material 1

Supplementary Material 2

## Data Availability

All NHANES data included in this study were publicly available at http://www.cdc.gov/nchs/nhanes/ and all GWAS data included in MR analysis were publicly available at https://gwas.mrcieu.ac.uk/.

## Abbreviations

BMD: Bone mineral density
BMI: Body mass index
DXA: Dual-energy x-ray absorptiometry
IGF-1: Insulin-like growth factor 1
IL-6: Interleukin-6
IVs: Instrumental variables
IVW: Inverse variance weighted
GWAS: Genome-wide association studies
LD: Linkage disequilibrium
MEC: Mobile examination center
MR: Mendelian randomization
NHANES: National Health and Nutrition Examination Survey
OR: Odds ratio
PIR: Poverty income ratio
SNPs: Single nucleotide polymorphisms
WHO: World Health Organization
WM: Weighted median
