# Supplementary material for: The association between osteoporosis and frailty: a cross-sectional observational study and mendelian randomization analysis
Source: J Orthop Surg Res. 2024 Jul 9;19:398. doi: 10.1186/s13018-024-04875-w (PMC11232274; doi:10.1186/s13018-024-04875-w)
Supplement: Supplementary file 1 — Supplementary Material 1 [file 13018_2024_4875_MOESM1_ESM.docx]

**Supplementary Figures:**

**Figure S1.** Scatter plot of the causal relationship between osteoporosis and Frailty index.

**
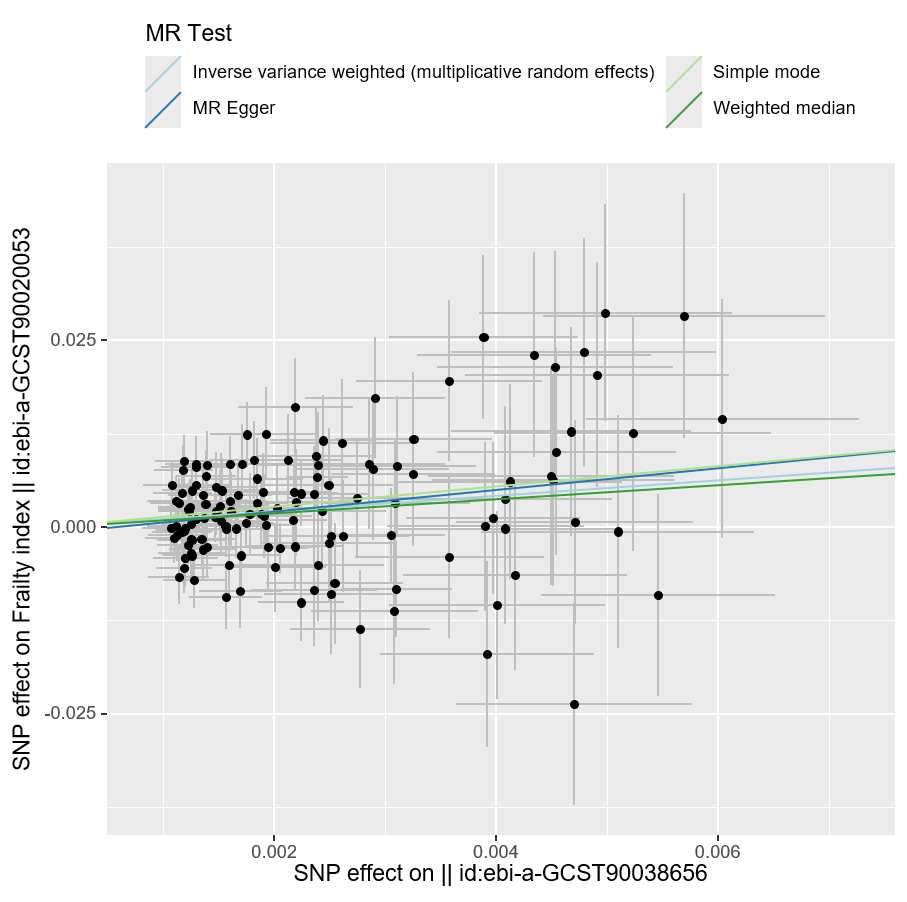
**

**Figure S2.** Forest plot of the causal relationship between osteoporosis and Frailty index.

**
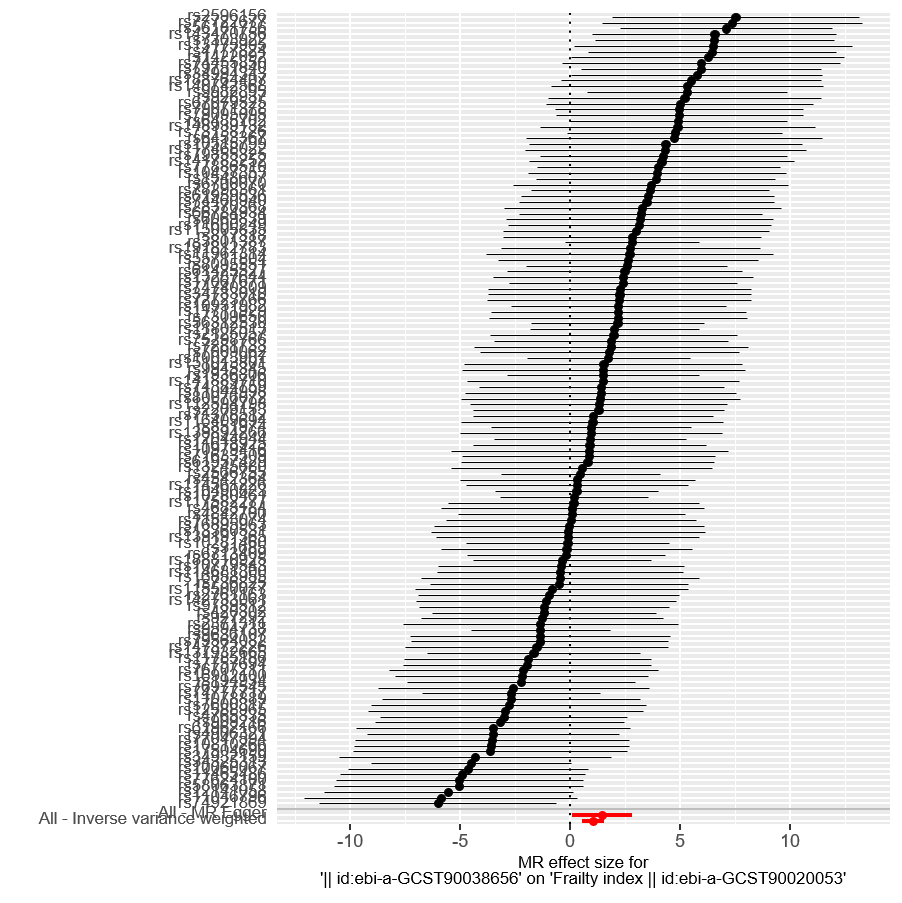
**

**Figure S3.** Leave-one-out plot of the causal relationship between osteoporosis and Frailty index.

**
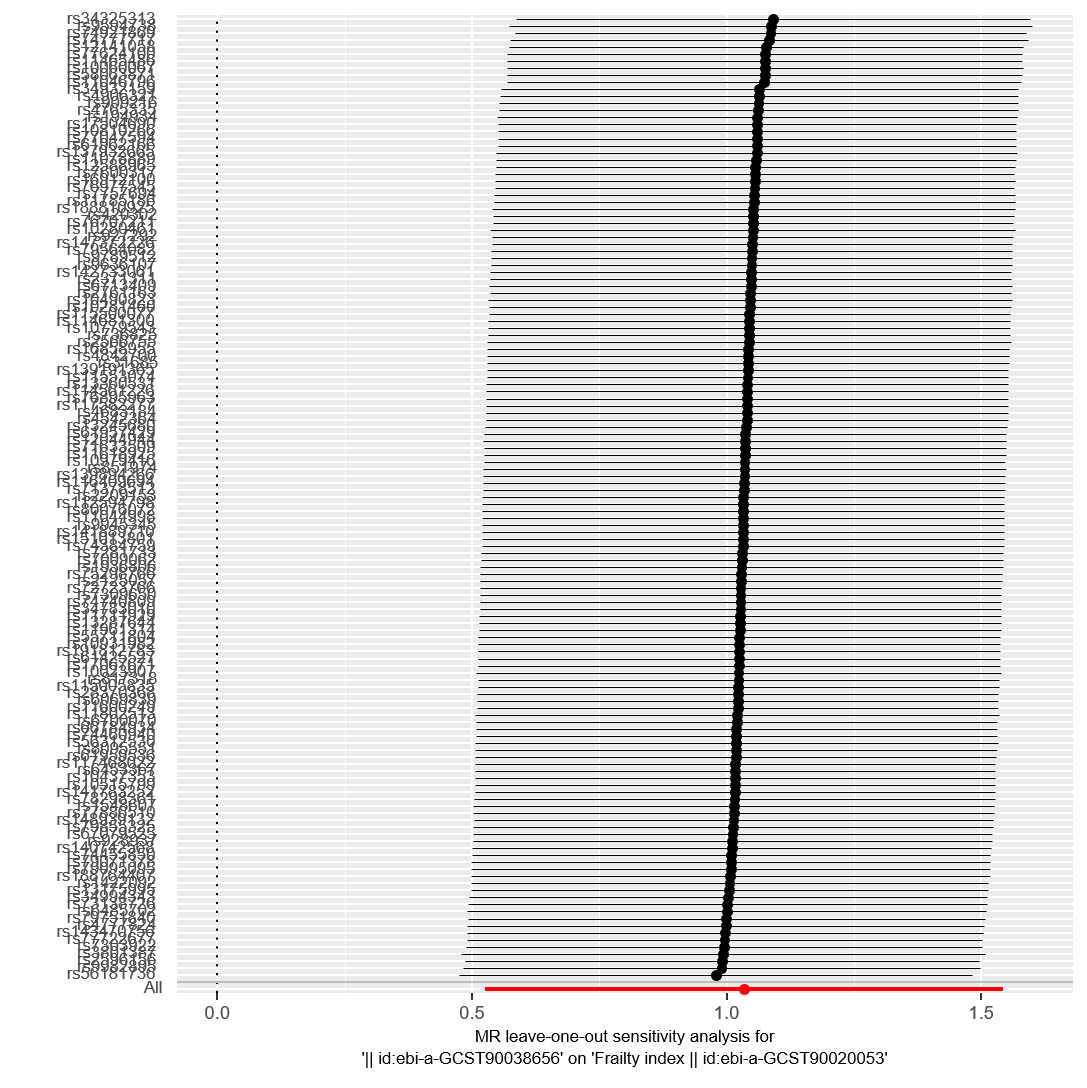
**

**Figure S4.** Funnel plot of the causal relationship between osteoporosis and Frailty index.

**
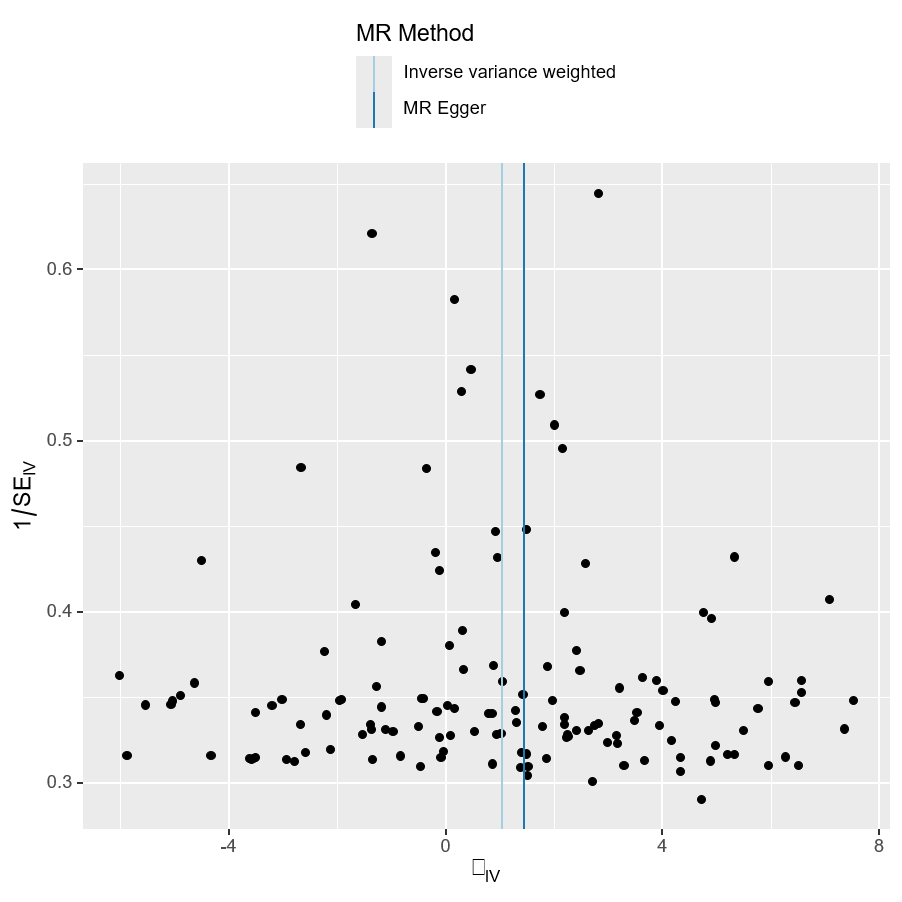
**

**Figure S5.** Scatter plot of the causal relationship between Frailty index and osteoporosis.

**
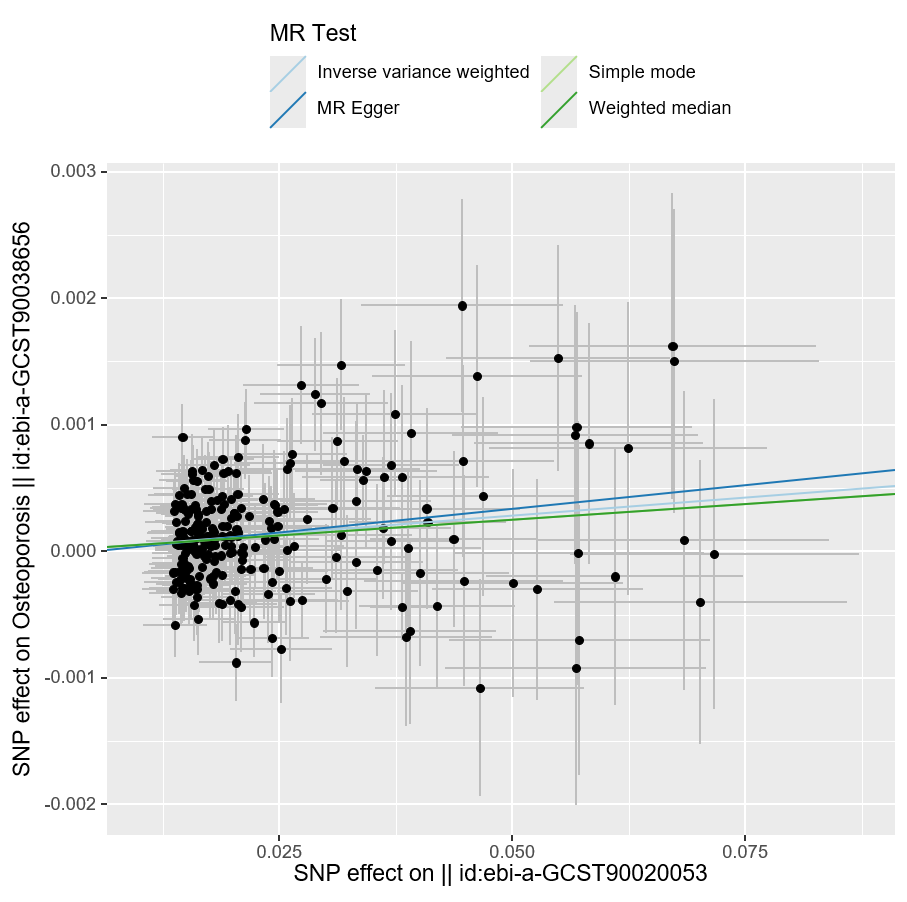
**

**Figure S6.** Forest plot of the causal relationship between Frailty index and osteoporosis.

**
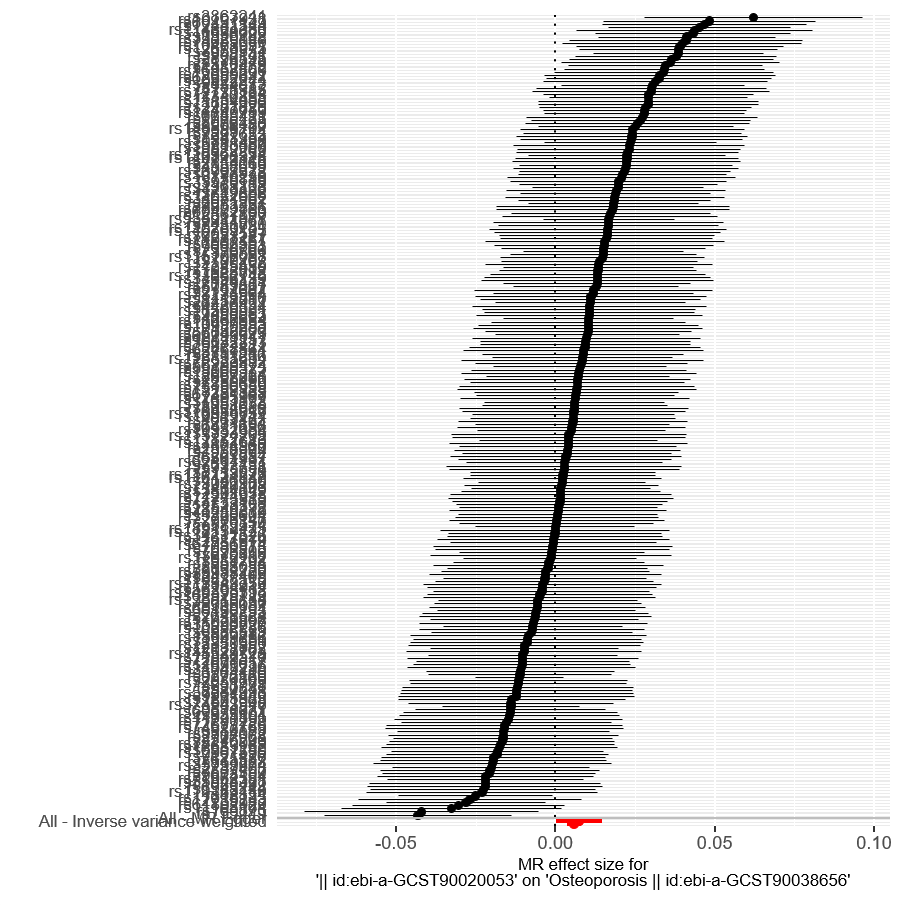
**

**Figure S7.** Leave-one-out plot of the causal relationship between Frailty index and osteoporosis.

**
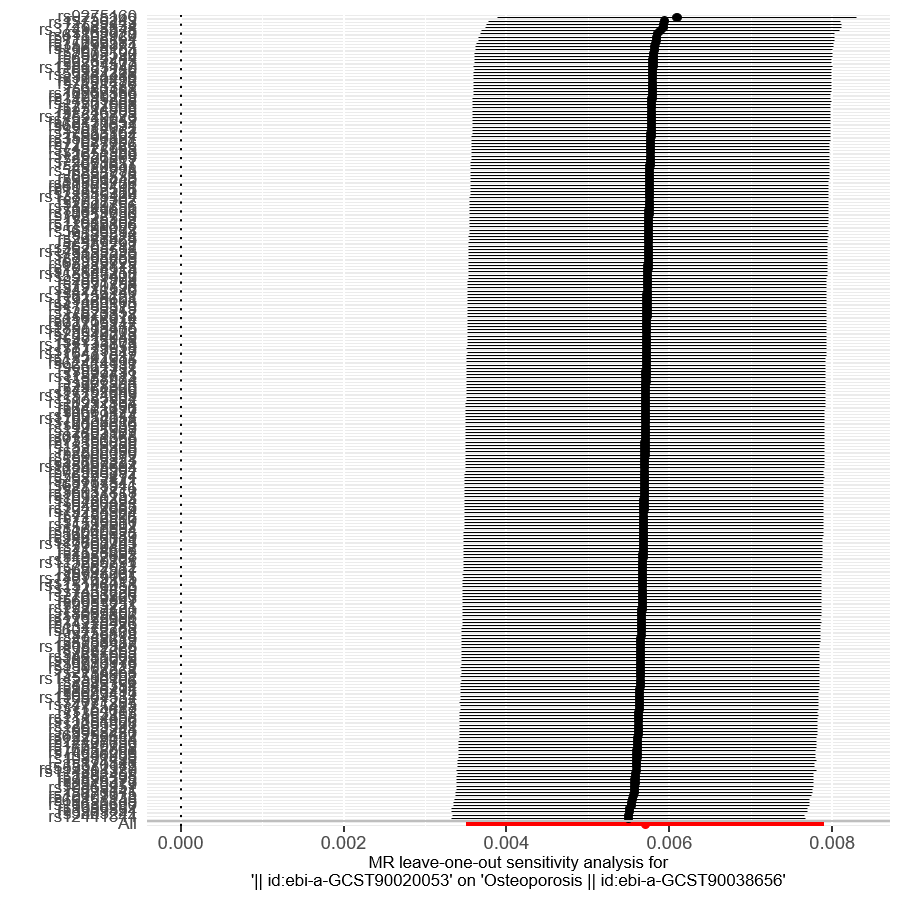
**

**Figure S8.** Funnel plot of the causal relationship between Frailty index and osteoporosis.

**
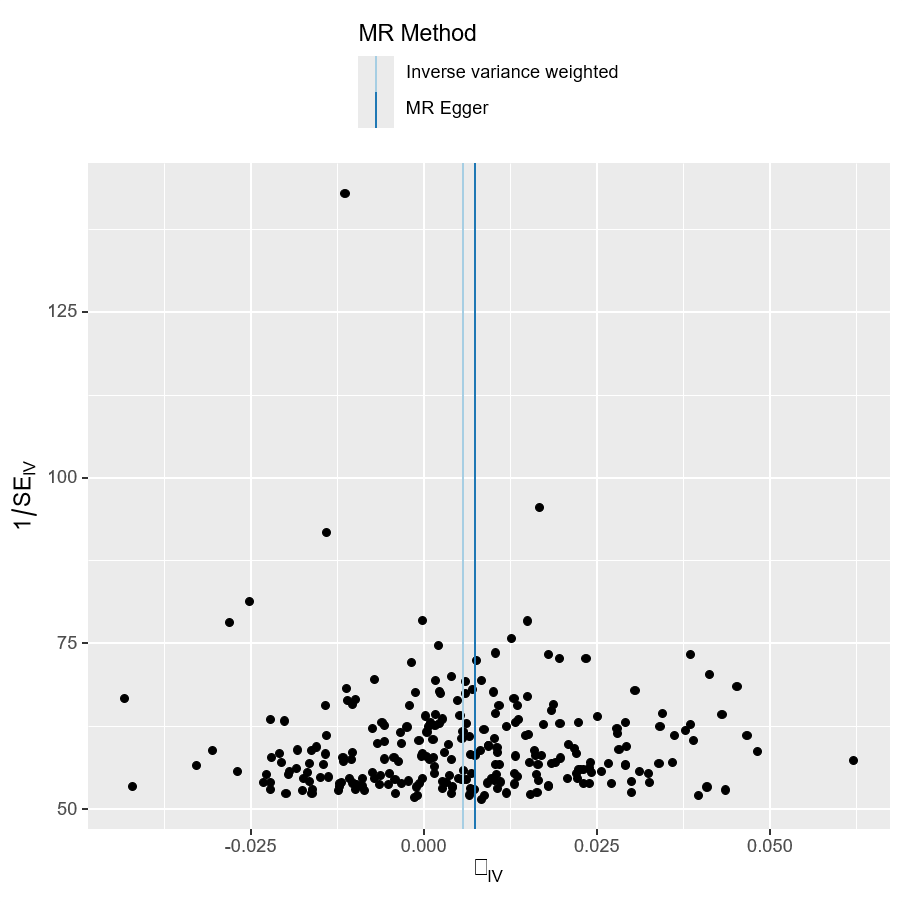
**
