# Supplementary material for: The association between osteoporosis and frailty: a cross-sectional observational study and mendelian randomization analysis
Source: J Orthop Surg Res. 2024 Jul 9;19:398. doi: 10.1186/s13018-024-04875-w (PMC11232274; doi:10.1186/s13018-024-04875-w)
Supplement: Supplementary file 2 — Supplementary Material 2 [file 13018_2024_4875_MOESM2_ESM.docx]

**Supplementary Table 1**
Variables in the 49-Item Frailty Index and Their Respective Scorings

| **Variable** | **Scoring** | |
| --- | --- | --- |
| **Cognition** |  | |
| 1. Experience confusion/memory problems | Yes = 1, No = 0 | |
| **Dependence** |  | |
| 2. Managing money | Difficulty = 1, No Difficulty = 0 | |
| 3. Stooping, crouching, kneeling | Difficulty = 1, No Difficulty = 0 | |
| 4. Lifting or carrying | Difficulty = 1, No Difficulty = 0 | |
| 5. House chore | Difficulty = 1, No Difficulty = 0 | |
| 6. Preparing meals | Difficulty = 1, No Difficulty = 0 | |
| 7. Standing up from armless chair | Difficulty = 1, No Difficulty = 0 | |
| 8. Getting in and out of bed difficulty | Difficulty = 1, No Difficulty = 0 | |
| 9. Using fork, knife, drinking from cup | Difficulty = 1, No Difficulty = 0 | |
| 10. Dressing yourself | Difficulty = 1, No Difficulty = 0 | |
| 11. Standing for long periods difficulty | Difficulty = 1, No Difficulty = 0 | |
| 12. Grasp/holding small objects | Difficulty = 1, No Difficulty = 0 | |
| 13. Attending social event | Difficulty = 1, No Difficulty = 0 | |
| 14. Push or pull large objects | Difficulty = 1, No Difficulty = 0 | |
| 15. Walking for a quarter mile difficulty | Difficulty = 1, No Difficulty = 0 | |
| 16. Walking up 10 steps difficulty | Difficulty = 1, No Difficulty = 0 | |
| **Depressive Symptoms** |  | |
| 17. Have little interest in doing things | Nearly every day = 1, More than half the days = 0.66, Several days = 0.33, Not at all = 0 | |
| 18. Feeling down, depressed, or hopeless | Nearly every day = 1, More than half the days = 0.66, Several days = 0.33, Not at all = 0 | |
| 19. Trouble sleeping or sleeping too much | Nearly every day = 1, More than half the days = 0.66, Several days = 0.33, Not at all = 0 | |
| 20. Feeling tired or having little energy | Nearly every day = 1, More than half the days = 0.66, Several days = 0.33, Not at all = 0 | |
| 21. Poor appetite or overeating | Nearly every day = 1, More than half the days = 0.66, Several days = 0.33, Not at all = 0 | |
| 22. Feeling bad about yourself | Nearly every day = 1, More than half the days = 0.66, Several days = 0.33, Not at all = 0 | |
| 23. Trouble concentrating on things | Nearly every day = 1, More than half the days = 0.66, Several days = 0.33, Not at all = 0 | |
| **Comorbidities** |  | |
| 24. Arthritis | Yes = 1, Suspect = 0.5, No = 0 | |
| 25. Thyroid problems | Yes = 1, Suspect = 0.5, No = 0 | |
| 26. Chronic bronchitis | Yes = 1, Suspect = 0.5, No = 0 | |
| 27. Cancer | Yes = 1, Suspect = 0.5, No = 0 | |
| 28. Congestive heart failure | Yes = 1, Suspect = 0.5, No = 0 | |
| 29. Coronary heart disease | Yes = 1, Suspect = 0.5, No = 0 | |
| 30. Angina | Yes = 1, Suspect = 0.5, No = 0 | |
| 31. Heart attack | Yes = 1, Suspect = 0.5, No = 0 | |
| 32. Stroke | Yes = 1, Suspect = 0.5, No = 0 | |
| 33. Blood pressure | Yes = 1, Suspect = 0.5, No = 0 | |
| 34. Diabetes | Yes = 1, Suspect = 0.5, No = 0 | |
| 35. weak/failing kidneys | Yes = 1, Suspect = 0.5, No = 0 | |
| 36. Urinary Leakage | Yes = 1, Suspect = 0.5, No = 0 | |
| **Hospital Utilization and Access to Care** |  | |
| 37. Self-rated health | Fair, poor = 1, Excellent, Very good, good = 0 | |
| 38. Health now compared with 1 year ago | Worse = 1, About the same, better = 0 | |
| 39. Overnight hospital patient in past year | Yes = 1, No = 0 | |
| 40. Frequency of health care use during past year | None = 0, 1–5 = 0,5, More than 5 = 1 | |
| 41. Number of prescribed medications | None = 0, 1–4 = 0.5, 5 and more = 1 | |
| **Physical Performance and Anthropometry** |  | |
| 42. Body mass index | <18.5, ≥30 = 1 25–<30 = 0.5 18.5–25 = 0 | |
| 43. Handgrip strength | MALE: For BMI ≤ 24, GS ≤ 29 For BMI 24.1–28, GS ≤ 30 For BMI >28, GS ≤ 32 = 1 | FEMALE: For BMI ≤ 23, GS ≤ 17 For BMI 23.1–26, GS ≤ 17.3 For BMI 26.1–29, GS ≤ 18 For BMI>29, GS ≤ 21 = 1 |
| **Laboratory Values** |  |  |
| 44. Glycohemoglobin (%) | 0%–5.7% = 0, >5.7% = 1 | |
| 45. Red blood cell count (million cells/μL) | M: 4.7–6.1 = 0, Other = 1 | F: 4.2–5.4 = 0, Other = 1 |
| 46. Hemoglobin (g/dL) | M: 13.5–18 = 0, Other = 1 | F: 12–16 = 0, Other = 1 |
| 47. Red cell distribution width (%) | 11.6–14.6 = 0, Other = 1 | |
| 48. Lymphocyte percent (%) | 20–40 = 0, Other = 1 | |
| 49. Segmented neutrophils percent (%) | 40–80 = 0, Other = 1 | |

BMI, Body mass index; GS, grip strength.
Sourced from *Hakeem F. F.; Bernabé E.; Sabbah W. Association Between Oral Health and Frailty Among American Older Adults. J Am Med Dir Assoc 2021, 22 (3), 559-563.e2. https://doi.org/10.1016/j.jamda.2020.07.023.*
